# Development of the Chicago Food Allergy Research Surveys: assessing knowledge, attitudes, and beliefs of parents, physicians, and the general public

**DOI:** 10.1186/1472-6963-9-142

**Published:** 2009-08-07

**Authors:** Ruchi S Gupta, Jennifer S Kim, Elizabeth E Springston, Jacqueline A Pongracic, Xiaobin Wang, Jane Holl

**Affiliations:** 1 Smith Child Health Research Program, Children's Memorial Hospital, Chicago, USA; 2 Institute for Healthcare Studies, Northwestern University Feinberg School of Medicine, Chicago, USA; 3 Division of Allergy and Immunology, Children's Memorial Hospital, Chicago, USA

## Abstract

**Background:**

Parents of children with food allergy, primary care physicians, and members of the general public play a critical role in the health and well-being of food-allergic children, though little is known about their knowledge and perceptions of food allergy. The purpose of this paper is to detail the development of the Chicago Food Allergy Research Surveys to assess food allergy knowledge, attitudes, and beliefs among these three populations.

**Methods:**

From 2006–2008, parents of food-allergic children, pediatricians, family physicians, and adult members of the general public were recruited to assist in survey development. Preliminary analysis included literature review, creation of initial content domains, expert panel review, and focus groups. Survey validation included creation of initial survey items, expert panel ratings, cognitive interviews, reliability testing, item reduction, and final validation. National administration of the surveys is ongoing.

**Results:**

Nine experts were assembled to oversee survey development. Six focus groups were held: 2/survey population, 4–9 participants/group; transcripts were reviewed via constant comparative methods to identify emerging themes and inform item creation. At least 220 participants per population were recruited to assess the relevance, reliability, and utility of each survey item as follows: cognitive interviews, 10 participants; reliability testing ≥ 10; item reduction ≥ 50; and final validation, 150 respondents.

**Conclusion:**

The Chicago Food Allergy Research surveys offer validated tools to assess food allergy knowledge and perceptions among three distinct populations: a 42 item parent tool, a 50 item physician tool, and a 35 item general public tool. No such tools were previously available.

## Background

Food allergy is a growing problem [1,2], affecting between 6–8% of children in the United States [3-5]. In fact, food has been shown to be the most common cause of childhood anaphylaxis [6], a potentially fatal reaction that can be prevented only by strict avoidance of allergenic foods [3,7,8] and results in an estimated 150 US deaths per year [9]. Accordingly, recognition of hidden food allergens is vital to the prevention of life-threatening episodes and death.

Food is ubiquitous and often plays a central role in many types of social gatherings. It is also frequently used as a reward or a symbol for celebration for young children. As such, food allergy concerns not only the families of affected children but also schools, restaurants, and airlines, to name a few. Due to the growing nature of the problem, more attention has been brought to the disease, though little is known about the knowledge, attitudes, and beliefs of food allergy among parents of children with food allergy, primary care physicians, and the general public. Each group, however, plays a critical role in the health and well-being of affected children.

The role of parents and families of children with food allergy is well-documented; the burden of risk assessment is placed on caregivers of food-allergic children and has been shown to adversely affect quality of life [7,10,11]. Primary care physicians, including pediatricians and family physicians, are often the first and sometimes the only clinicians to diagnose and manage food allergy in a child. The general public plays a part as well, as they often interact with young children at restaurants, entertainment facilities, and schools. Many lifestyles now depend on food prepared away from home [12], and 76% of food allergy deaths follow food consumption outside of the home [13]. Furthermore, approximately 18% of children with food allergy have at least 1 reaction at school within a 2-year period [8].

In spite of the latter, there do not appear to be any validated, population-specific survey instruments designed to assess perceptions and understanding of food allergy. Without a cure, such instruments are necessary to characterize baseline knowledge, to develop effective education, advocacy, and prevention strategies, and to measure the impact of these strategies. The purpose of this paper is to detail the development of the Chicago Food Allergy Research Surveys, 3 validated survey instruments to assess food allergy knowledge, attitudes, and beliefs of (1) parents of children with food allergy, (2) pediatricians and family physicians, and (3) the general public.

### Overview of development

**Figure 1:**
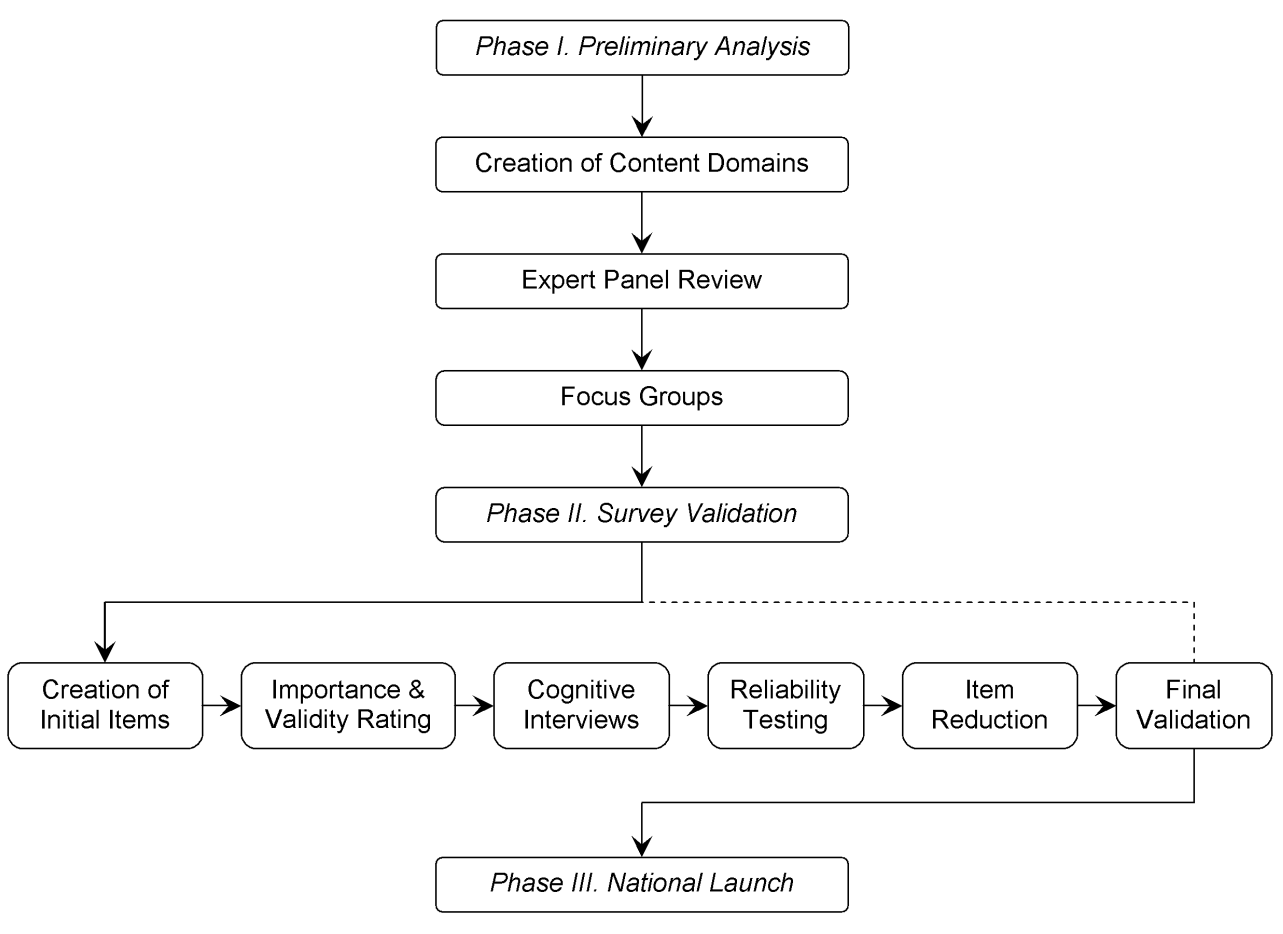
**Stages in the development of survey instruments to assess food allergy knowledge, attitudes, and beliefs of parents, physicians, and the general public**.

Figure 1 outlines the process undertaken in the development of each validated survey instrument. Preliminary analysis (Phase I) consisted of a review of published literature and internal collaboration to aid in the creation of initial content domains. Initial domains were then submitted to a group of experts in the field of food allergy for review, revision, and approval. Focus groups were conducted for each survey population to identify emerging themes within content domains. Survey validation (Phase II) followed, with utilization of focus group themes in the construction of initial survey items. Initial items were submitted to the expert panel to assess the importance and face validity of each item. Upon item revision and approval, cognitive interviews were conducted with members of each survey population to ensure the understandability. Items were then subject to reliability testing, to account for the consistency of participant response, followed by item reduction, to remove superfluous and nonessential survey items. Final validation was conducted in tandem with the national administration of the survey (Phase III), using a soft launch of 150 responses to assure the overall validity of each instrument.

## Methods

Participation in each phase of survey development and administration was anonymous. Written, informed consent was obtained from participants in the focus groups and cognitive interviews. Written consent was waived for the remaining phases of development and administration. Participants were informed that submission of the survey indicated consent to participate and were told that results would not be linked to any identifying information.

The development and administration of each survey instrument was approved by the Institutional Review Boards of Children's Memorial Hospital and Northwestern University Feinberg School of Medicine.

### Phase I: Preliminary analysis

#### Creation of content domains

The survey development process began with a review of previous food allergy literature to better understand important knowledge areas as well as current food allergy attitudes and beliefs. Informal discussions were also held with local parents of children with food allergy, physicians, and the general public.

A working group of parents, physicians, members of the general public, and survey researchers was established. Based on review of current literature and expert opinion, current topics related to food allergy knowledge, attitudes, and beliefs were identified. The topics were then assembled into similar groups, and content domains were formulated to encompass the dimensions of the Health Belief Model [14,15]. After thorough review, a set of 9 preliminary domains was identified, meant to embody relevant issues relating to food allergy knowledge, attitudes, and beliefs.

#### Expert panel review

An expert panel of 9 individuals was assembled, comprised of community pediatricians, two pediatric allergists with expertise in food allergy, survey researchers, a leader of the largest US food allergy advocacy network, and a parent of two food-allergic children who founded of a local parent support group. The panel was asked to review the preliminary content domains and to verify representation of all relevant food allergy topics. The panel was also encouraged to suggest inclusion of unrepresented areas, combination of overlapping domains, and deletion of nonessential domains. Following the panel's review, participants received an honorarium.

The working group reviewed the panel's suggestions and developed a final set of eight content domains: (1) definition and diagnosis, (2) symptoms and severity, (3) triggers and environmental risk, (4) perceptions of susceptibility and prevalence, (5) stigma and acceptability, (6) perceptions of quality of life, (7) treatment and utilization of healthcare, and (8) policy issues. These domains formed the framework from which questions were developed for the focus group protocol.

#### Focus groups

Focus groups were held to understand current knowledge under each domain. Three focus groups of parents (2 with mothers, 1 with fathers), 2 of physicians (1 with pediatricians, 1 with family physicians), and 2 of the general public (1 high-income, 1 low-income group) were conducted. All focus groups lasted 1–2 hours and were audio-taped and transcribed. Focus group locations were selected for participants' convenience and an honorarium was distributed to each individual upon conclusion of the eventt.

Investigators experienced and trained in facilitating focus groups led the discussions. Standard moderation techniques were used throughout [16]. Focus group participants received equal time for responding to questions. A standardized protocol of open-ended questions was used for each group with minor variations by survey population. Focus groups continued until all discussion on pertinent topics was exhausted.

Upon review of focus group transcripts, a coding scheme was developed. A constant comparative method was used to identify emerging themes [17,18]. Coding was facilitated by a qualitative data analysis software program, *Atlas.ti *(Atlas.ti; Berlin, Germany). At least 2 reviewers independently coded each transcript; the codes were then reconciled to produce a single coded transcript. Final codes were carefully reviewed by the working group.

### Phase II: Survey validation

#### Creation of initial items

The coded results from the focus groups informed the creation of domain-specific items to populate each survey instrument. A survey item for each unique code was created for a given domain. Each item was developed to: 1) signify the thought presented in the coded transcript, 2) represent the particular domain, 3) be clear and succinct, 4) avoid redundancy with other items, and 5) have a true/false/I don't know (T/F/IDK), multiple choice (MC) or Likert scale (LS) response. Additionally, to encourage comparison across populations, care was taken to incorporate items shared among survey instruments. Special attention was paid during sequential stages to any additions, modifications, or deletions of shared items.

After the creation of an initial item set, each member of the working group participated in a process of review and revision. A final set of 86 parent items, 65 physician items and 52 general public items were developed to represent the content domains. These items were then submitted to the expert panel to assess importance and face validity.

#### Importance and validity rating

Members of the expert panel were sent a spreadsheet containing items arranged by domain for each survey population. The panel was asked to rate both the importance

(0–2, 0 = not important, 2 = very important) and face validity (invalid = 0, valid = 1) of each item. Members were also asked to submit qualitative comments, develop additional items as needed, and propose improvements to existing items. An honorarium was distributed following the return completed spreadsheets for each survey population.

For each item, an average importance and face validity score was calculated from the expert panel ratings. The survey items were then rank-ordered by average importance score, ranging from 0–2. The working group reviewed the rank-ordered scores by domain, with a score from 0–1 signaling a candidate for deletion or modification; revisions were also made to improve items with scores less than 2. The group incorporated the panel's face-validity ratings, comments, suggestions, and proposed revisions into this process. For example, the parent statement "Most pediatricians know enough about food allergy (LS)," received a significance score of 0.8 and a face validity score of 0.4. The panel indicated that the statement was too subjective to be of value and the item was removed. Alternatively, the panel suggested inclusion of the parent statement "I have been frustrated because different doctors have told me different things about my child's food allergy (LS)," which was subsequently added.

#### Cognitive interviews

Cognitive interviews were held to assess the clarity and understandability of each item following expert panel review. Attention was also paid to survey details on the whole, including length, format, and general impressions. Ten individuals from each survey population were recruited to spend 1–2 hours reviewing items one-on-one with a member of the working group. Participants from the parent group were identified through local support groups, such as Mothers of Children Having Food Allergy. Physicians were targeted at area hospitals and clinics. Members of the general public were recruited evenly from a local elementary school and from the waiting room of a low-income pediatric clinic. Discussions were audio-taped and an honorarium was distributed at the interview's conclusion.

Participants were instructed to read and complete the survey, and enquiries were made as to its overall length and clarity. Participants were then asked to rate both the understandability (5 point LS, 1 = easy, 5 = hard) and domain relevance (i.e. "Does this item belong under the heading [insert content domain]?" no = 0, yes = 1) of each item. Participants were also asked to interpret items in their own words and to make suggestions for potential revisions. To gauge health literacy, parents and members of the general public were asked to complete the Rapid Estimate of Adult Literacy in Medicine [19].

For each item, average understandability and domain relevance scores were calculated from participant ratings. The working group reviewed the scores by domain and incorporated comments, suggestions, and proposed revisions into the decision-making process. As a rule of thumb, items with an average understandability score greater than 1 (i.e. 'easy'), were flagged for modification or deletion. For example, the general public statement "It is possible to grow out of a food allergy (T/F/IDK)," received an understandability rating of 1.3. The concept of tolerance was not well communicated, and, based on participant suggestion, the item was revised to read "Food allergies can go away as a person gets older." The general public statement "A shot of adrenaline or epinephrine is used to treat a serious food allergy reaction (T/F/IDK)," received an understandability rating of 2.6. Unlike the previous example, comments indicated that rating was not a consequence of poor-wording, but rather the result of subject matter beyond the scope of common knowledge. Therefore, the item was discarded.

#### Reliability testing

From the items revised following cognitive interviews, reliability testing was conducted to ascertain response consistency over a brief time interval. For ease of administration, a web-based survey was developed and utilized from this point forward. Participants were asked to complete the online survey twice (test, re-test) with the second round following 1 week after submission of the initial response.

Parents of children with food allergy were recruited by way of advertisement in health clinics, schools, activity centers, and child care centers; parents were also targeted through local support groups. Pediatricians and family physicians, as well as members of the general public, were recruited through local clinics and hospitals. A minimum of 10 participants was required for each survey. Response rates were high, with 91% of parents (n = 10), 100% of physicians (n = 12), and 81% of general public recruits (n = 13) completing the survey twice. An honorarium was distributed upon re-test completion.

To determine the reliability of data from test to re-test, a bright-line was drawn at 45% response variation for objective items and 50% response variation for LS items. Items that resulted in a degree of change at or beyond these thresholds were points of discussion and subject to revision. For example, the general public statement "Food allergies happen when the body considers food to be harmful (T/F/IDK)," resulted in a 46% variation in responses between test and re-test. Upon review, it was determined that the variation was a consequence of the question's lack of clarity rather than the reliability of the participant's response. Participants were contacted and asked to interpret the question; after consideration of their feedback, the questions was changed to read "An allergic reaction can happen when the body considers food to be harmful (T/F/IDK)," and highlighted for further review following the conclusion of item reduction.

#### Item reduction

A larger sample pool completed each survey following revisions from reliability testing. Item reduction was employed to avoid floor and ceiling effects and to ensure sufficient response distribution across various sectors of the population. Additionally, attention was paid to the importance and novelty of the data generated from each item. The overall length of each tool was also a consideration at this stage of development.

Participants from the parent group were obtained via the recruitment methods outlined for reliability testing. Further, parents of low-income patients from a pediatric allergy clinic were contacted by mail and asked to complete and return a paper-based survey. Clinicians were targeted through local chapters of professional organizations, such as the American Academy of Pediatrics, in addition to the methods previously outlined. Members of the general public were recruited through web posts on local sites, such as Craigslist ; a paper-based survey was also administered to adults recruited from the waiting room of a low-income pediatric clinic. A minimum of 50 participants was required for each survey. Response rates varied, with 72% of parents (n = 57), roughly 50% of physicians (n = 62), and 89% of general public recruits (n = 54) completing the survey. An honorarium was distributed upon completion.

A response ceiling of 80% for objective survey items and 90% for combined LS items (e.g. selection of either 'strongly disagree' or 'disagree') was set. Survey items producing a response cluster at or above the latter were subject to deletion; examples are detailed in Table 1.

**Table 1:** Examples of deletions made during item reduction.

**Item**	**% Respondents in Agreement (SD)**	**Response Selected**
*Parent Survey*		

"Two children who are allergic to the same food will always have the same reaction when they eat that food."	96.5 (0.19)	False
"Having an EpiPen or Twinject is important for children with severe food allergies."	98.3 (0.13)	Strongly Agree or Agree

*Physician Survey*		

"Genetics is a risk factor for the development of food allergy."	94.0 (0.44)	True
"The rate of parent-reported food allergy is higher than clinically diagnosed food allergy."	88.0 (0.34)	True

*General Public Survey*		

"For a person with a walnut allergy, eating pretzels from a jar that had walnuts in it before can cause an allergic reaction."	84.2 (0.61)	True
"Food labels help people with food allergies avoid foods that they are allergic to."	85.9 (0.56)	Strongly Agree or Agree

#### Final validation

Final validation was conducted in tandem with national administration of the survey, using a soft launch of the first 150 responses. The purpose of the soft launch was to identify lingering issues with the intent to delete problematic items of questionable validity.

Parents were recruited via targeted web-posts on food allergy resource pages, such as the Food Allergy Project's website . Additionally, support groups nationwide were contacted and agreed to recruit parents of children with food allergy, utilizing list-serves and monthly newsletters. Word-of-mouth also proved to be a powerful tool at this stage of development. An honorarium was distributed to the first 1500 respondents. Consultants were employed in both the recruitment of physicians (Redi-Data; Fairfield, NJ USA) and members of the general public (e-Rewards Market Research; Dallas, TX USA). A portion of the AMA master file was purchased, containing contact information for 4500 pediatricians and 1500 family physicians; a targeted e-broadcast was then deployed with a direct link to the survey. An honorarium was distributed to the first 400 respondents. The general public was recruited by way of a commercial vendor specializing in national sampling; incentives were determined and distributed by the vendor to the first 2000 respondents. For each survey, participation was contingent upon criteria designed to ensure a representative sample of the target population.

Few modifications were made during final validation. However, several comments were made by parents of children with food allergy regarding the statement, "A peanut-allergic child can have an allergic reaction from the smell of peanut butter (T/F/IDK)." Parents disputed the correct response ("false") and it was agreed that, at the very least, the statement was unclear and the reliability of the data was questionable. The shared item, asked of each population group, was discarded from all surveys.

## Results

### Phase III: National launch

Table 2 summarizes item status for each survey instrument throughout Phases I and II. Conclusion of the latter phases resulted in 3 validated survey instruments, summarized in Table 3. The instruments include a 42 item parent tool [see Additional File 1], a 50 item physician tool [see Additional File 2], and a 35 item general public tool [see Additional File 3]. Each survey was administered nationally as described above. Data was obtained from 2148 members of the general US population. Data collection for the parent and primary care physician surveys is ongoing; thus far, responses have been received from over 3000 parents of children with food allergy and over 400 pediatricians and primary care physicians.

**Table 2:** Item status at the conclusion of survey development stages

	**Item Status**			
	
**Stage of Development**	**Added**	**Modified**	**Discarded**	Total*
*Parent Survey*				

Creation of Initial Items				84
Importance/Validity Rating	14	25	15	83
Cognitive Interviews	10	33	28	65
Reliability Testing	6	20	6	65
Item Reduction			22	43
Final Validation			1	42

*Physician Survey*				

Creation of Initial Items				65
Importance/Validity Rating	18	23	9	74
Cognitive Interviews	1	31	8	67
Reliability Testing	1	6		68
Item Reduction		8	17	51
Final Validation			1	50

*General Public Survey*				

Creation of Initial Items				52
Importance/Validity Rating	5	11	6	51
Cognitive Interviews	2	12	7	46
Reliability Testing		1		46
Item Reduction		1	10	36
Final Validation			1	35

*Total indicates number remaining at *conclusion* of given stage

**Table 3:** Characteristics of the final, validated survey instruments

	**Validated Survey**		
	
**Details**	**Parent**^1^	**Physician**	**General Public**
Total items	42	50	35
True/False/I don't know	8	16	16
Multiple Choice	11	24	6
Likert scale	23	10	13
Knowledge-based items	15	39	19
Shared items^2^	19	22	15
Estimated completion time	15 minutes	<15 minutes	<10 minutes

^1 ^The parent survey also includes a validated child-grid for optional use to obtain relevant medical information regarding the participant's children with and without food allergy. Completion of the survey requires roughly 20 minutes with inclusion of the child-grid.

^2 ^Items are shared accordingly: Parent & physician tools–16 items; parent & general public tools–9 items; physician & general public tools–12; parent, physician & general public tools–6 items.

The development and validation of the Chicago Food Allergy Research Surveys demonstrates the necessity of careful and deliberate activity in the creation of instruments to measure knowledge accurately and reliably. Significant changes were made at each stage of the process (Table 2) to ensure inclusion and retention of items with face validity and good performance characteristics. Similar methodology has been used by other research groups to generate effective knowledge tools with like objectives and applications [20,21]. Three validated survey tools have been created which are representative of 8 significant content domains established to assess food allergy knowledge, attitudes, and beliefs.

## Discussion

The purpose and utility of each validated survey instrument is multifold. The parent survey provides insight into misconceptions surrounding food allergy while also identifying how the knowledge, attitudes, and beliefs of physicians and general public impacts parents and children with food allergy directly. Clinically, administration of the tool to families of food-allergic patients provides a way for primary care providers to identify areas of difficulty prior to consultation.

The physician instrument itself is also of clinical value. Data from the national launch will be used to assess the knowledge of pediatricians and family physicians in the US, particularly regarding diagnosis and management of children with food allergy. Identification of common misconceptions will be used in the development of an interactive, web-based, educational tool for physicians. The physician survey may also be used in pre/post evaluations of food allergy aptitude in continuing medical education courses. Similarly, the tool may be used to gauge the food allergy knowledge of physicians-in-training.

The general public instrument is a powerful tool in public awareness. Increasing prevalence [1,2] has brought recent attention to childhood food allergy–organized food allergy support groups exist in many states in the US and large national food allergy organizations, such as the Food Allergy and Anaphylaxis Network, are promoting increased knowledge and awareness among the public. Large food allergy organizations, local support groups, and physician organizations may find the general public survey useful for obtaining baseline assessments, determining community attitudes towards food allergy, and evaluating the effectiveness of educational campaigns and courses.

There are limitations to the study design that must be highlighted. While systematic effort was made to ensure that the surveys encompass all areas of food allergy knowledge, it is possible that topics were overlooked. Preliminary validation was limited to the Chicago area. However, the expert panel was comprised of a far-spread group of nationally-recognized professionals and the web-based stages of development included participants from across the US. Specifically, final validation included representatives from every US Census region. Finally, the surveys were developed for a wide audience. If a given survey is intended to be used in a more specialized manner–for instance, in the education of child care employees–addition and validation of more specific items may complement the existing tool.

## Conclusion

The Chicago Food Allergy Research Surveys for parents, primary care physicians, and the general public will assist in understanding the current state of food allergy knowledge and in the development of educational tools and awareness campaigns to address dangerous misconceptions in the US and abroad. For example, data from the national administration of the general public survey indicates a need for concerted education efforts regarding the distinction between food allergy and food intolerance, the absence of a cure, and current means to treat food allergy; the data also point towards the necessity of educating parents of school-aged children about the importance of school policies to keep food-allergic children safe [22]. Preliminary analysis of responses from parent and primary care providers similarly suggests localized strengths and weaknesses in participants' knowledge of food allergy.

With no cure for food allergy, adequate knowledge of common allergenic foods, signs and symptoms, and appropriate treatments are essential for the prevention of allergic reactions, anaphylaxis, and death. We believe that the surveys will help improve the food allergy knowledge of parents of children with food allergy, physicians, who often diagnose and treat food allergy, and the general public, who often come in contact with food-allergic children. This will in turn improve the lives of children and families affected by food allergy.

## Competing interests

The authors declare that they have no competing interests.

## Authors' contributions

RSG developed the initial design of the study, participated in each stage of development and validation, and drafted/edited the manuscript. JSK developed the initial design of the study, participated in each stage of development and validation, and drafted/edited the manuscript. EES participated in survey validation, coordinated recruitment and data collection, developed the tables and drafted/edited the manuscript. JAP, XW and JH developed the initial design of the study, oversaw each stage of development and validation, and edited the manuscript. All authors read and approved the final manuscript.

## Pre-publication history

The pre-publication history for this paper can be accessed here:



## Supplementary Material

Additional file 1 **The Chicago Food Allergy Research Survey for Parents of Children with Food Allergy**. Final validated survey instrument for parents of food-allergic children; tool is available to all clinicians/researchers in order to (1) assess food allergy knowledge, (2) identify attitudinal barriers towards food allergy, and (3) develop targeted and effective interventions to improve the lives of food-allergic children and families. Click here for file

Additional file 2 **The Chicago Food Allergy Research Survey for Primary Care Physicians**. Final validated survey instrument for pediatricians and family physicians; tool is available to all clinicians/researchers in order to (1) assess food allergy knowledge, (2) identify attitudinal barriers towards food allergy, and (3) develop targeted and effective interventions to improve the lives of food-allergic children and families. Click here for file

Additional file 3 **The Chicago Food Allergy Research Survey for the General Public**. Final validated survey instrument for the general public; upon publication, tool is available to all clinicians/researchers in order to (1) assess food allergy knowledge, (2) identify attitudinal barriers towards food allergy, and (3) develop targeted and effective interventions to improve the lives of food-allergic children and families. Click here for file
