# Supplementary material for: Development of the Chicago Food Allergy Research Surveys: assessing knowledge, attitudes, and beliefs of parents, physicians, and the general public
Source: BMC Health Serv Res. 2009 Aug 7;9:142. doi: 10.1186/1472-6963-9-142 (PMC2736935; doi:10.1186/1472-6963-9-142)
Supplement: Additional file 1 — The Chicago Food Allergy Research Survey for Parents of Children with Food Allergy. Final validated survey instrument for parents of food-allergic children; tool is available to all clinicians/researchers in order to (1) assess food allergy knowledge, (2) identify attitudinal barriers towards food allergy, and (3) develop targeted and effective interventions to improve the lives of food-allergic children and families. [file 1472-6963-9-142-S1.pdf]

The Chicago Food Allergy Research Surveys for Parents of Children with Food Allergy

The following survey is part of a study being conducted by researchers at Children's Memorial Hospital and Northwestern University Feinberg School of Medicine in Chicago, Illinois. The goal of this survey is to assess the knowledge, attitudes and beliefs around food allergy of ***parents of children with food allergies****.*

Before beginning the survey, please answer the following questions:

1. Do you have at least one child under the age of 18 with a food allergy that has been diagnosed by a doctor?

   ❐ Yes

   ❐ No ➔ We're sorry, but you are not eligible for this survey. Thank you for your interest.

2. Are you a member of a food allergy support group? ❐ Yes ❐ No

3. In which state do you live?
   State: ______________________

Please mark one box for each statement.

	TRUE	FALSE	I DON'T KNOW
1. Food allergy involves the immune system.	☐	☐	☐
2. The only way to know that your child is allergic to a food is with a medical test.	☐	☐	☐
3. Eczema may be the first sign of having a food allergy.	☐	☐	☐
4. Asthma is an important risk factor for severe anaphylaxis.	☐	☐	☐
5. Foods eaten by a mother can be passed to her child through her breast milk.	☐	☐	☐
6. Food additives (like citric acid, colorings, starch) are common food allergens.	☐	☐	☐
7. Teenagers are at higher risk for fatal food allergy compared to younger children.	☐	☐	☐
8. Taking a daily antihistamine (like Benadryl or Claritin) can prevent food allergy reactions.	☐	☐	☐

9. Which of the following contain food ingredients that could cause an allergic reaction? Mark all that apply.
   - ❒ Play-doh
   - ❒ Medicines
   - ❒ Toothpaste
   - ❒ Lotions & creams

10. A boy with a milk allergy accidentally drank some milk. Please mark which of the following could be a sign of a food allergy reaction. Mark all that apply.
    - ❒ After 2 days he gets hyperactive, cranky, & complains of headaches
    - ❒ After 1 hour he has hives on his face and chest
    - ❒ Immediately his tongue swells and he has trouble breathing
    - ❒ He has a stuffy nose that won't go away for weeks

11. Which of the following are the three most common allergies in children? Mark three answers.
    - ❒ Egg
    - ❒ Wheat
    - ❒ Milk
    - ❒ Peanut
    - ❒ Tree nuts *(almonds, walnuts, pecans, cashews)*
    - ❒ Shellfish *(shrimp, lobster, crab)*

12. Which age group is the most likely to have food allergies?
    - ❒ 0 - 5 years
    - ❒ 6 - 10 years
    - ❒ 11 years or older
    - ❒ I don't know

13. What percent of children with milk allergies develop tolerance (or outgrow the allergy)?
    - ❒ Less than 1%
    - ❒ Between 1 - 25%
    - ❒ Between 26% - 50%
    - ❒ Between 51% - 75%
    - ❒ Greater than 75%
    - ❒ I don't know

14. What percent of children with peanut allergies develop tolerance (or outgrow the allergy)?
    - ❒ Less than 1%
    - ❒ Between 1 - 25%
    - ❒ Between 26% - 50%
    - ❒ Between 51% - 75%
    - ❒ Greater than 75%
    - ❒ I don't know

15. Where is the best place to use an EpiPen or Twinject (injectable epinephrine)?
    - ❒ Upper arm
    - ❒ Outer thigh
    - ❒ Buttock
    - ❒ I don't know

➢ ***Please continue to the next page.***

Do your have a significant other, such as a spouse or partner?

❒ No ➡ **Please skip to the next page.**

❒ Yes ➡ **Please continue to the next question.**

How does having a child with food allergy affect your relationship with your husband, wife, boyfriend, girlfriend, or partner? **Please mark one box for each statement.**

	Strongly Disagree	Disagree	Neither Agree nor Disagree	Agree	Strongly Agree	Does Not Apply
16. My child's food allergy causes a strain on my marriage/relationship.	☐	☐	☐	☐	☐	☐
17. He/she and I do not agree on how to take care of our child's food allergy.	☐	☐	☐	☐	☐	☐
18. I pay more attention than him/her in keeping our child away from the allergenic food(s).	☐	☐	☐	☐	☐	☐
19. I worry more than him/her about our child's food allergy.	☐	☐	☐	☐	☐	☐
20. He/she thinks that I exaggerate the seriousness of our child's food allergy.	☐	☐	☐	☐	☐	☐
21. His/her career has suffered because of our child's food allergy.	☐	☐	☐	☐	☐	☐

➢ ***Please continue to the next page.***

Please mark one box for each statement.

	Strongly Disagree	Disagree	Neither Agree nor	Agree	Strongly Agree	Does Not Apply
22. I have relatives who do not accommodate my child's food allergy.	☐	☐	☐	☐	☐	☐
23. My child's friends treat my child differently because of his or her food allergy.	☐	☐	☐	☐	☐	☐
24. I have experienced hostility from parents of children without food allergies when I have asked them to make accommodations for my child's food allergy.	☐	☐	☐	☐	☐	☐
25. The way I deal with my child's food allergy affects the way my child deals with his or her own food allergy.	☐	☐	☐	☐	☐	☐
26. Having a child with a food allergy affects my other children's daily lives.	☐	☐	☐	☐	☐	☐
27. Having a child with a food allergy causes money problems for my family.	☐	☐	☐	☐	☐	☐
28. My career has suffered because of my child's food allergy.	☐	☐	☐	☐	☐	☐
29. I have been frustrated because different doctors have told me different things about my child's food allergy.	☐	☐	☐	☐	☐	☐
30. Most people know the symptoms of food allergy.	☐	☐	☐	☐	☐	☐
31. Most people take food allergy seriously.	☐	☐	☐	☐	☐	☐

32. Have you or your significant other made career changes becuase of your child's food allergy? Mark all that apply.
❒ Yes, I have ❒ Yes, my significant other has
❒ No

33. Have you or your significant other chosen not to work outside the home because of your child's food allergy? Mark all that apply.
❒ Yes, I have ❒ Yes, my significant other has
❒ No ❒ Not applicable

➢ ***Please continue to the next page.***

Please mark one box for each statement.

	Strongly Disagree	Disagree	Neither Agree nor Disagree	Agree	Strongly Agree
34. I feel confident that the staff in my child's school or daycare is knowledgeable in the management of food allergy emergencies.	☐	☐	☐	☐	☐
35. Children with food allergies should be allowed to carry an EpiPen or Twinject with them at school.	☐	☐	☐	☐	☐
36. In schools, an EpiPen or Twinject should be kept only in nurse's offices.	☐	☐	☐	☐	☐
37. Schools should ban all products with nuts.	☐	☐	☐	☐	☐
38. Schools should have an adult with experience or training in food allergy always available during lunch periods, parties, and field trips.	☐	☐	☐	☐	☐
39. Food labeling laws have decreased the choice of foods that my child can eat.	☐	☐	☐	☐	☐
40. More government money should go toward research about food allergy.	☐	☐	☐	☐	☐

41. Which of the following do you think is the most important to help people with food allergies?

- ❒ Develop a cure for food allergy
- ❒ Improve the treatments of food allergy
- ❒ Identify the causes of food allergy
- ❒ Promote school education programs about food allergy
- ❒ Promote public awareness campaigns about food allergy

42. Which of the following resources have you used for food allergy education or support? Mark all that apply.

- ❒ American Academy of Pediatrics (AAP)
- ❒ Food Allergy & Anaphylaxis Network (FAAN)
- ❒ Internet/websites
  **Like www.foodallergyproject.com or *www.webmd.com*
- ❒ Government agencies
  **Like Food and Drug Administration (FDA), Centers for Disease Control (CDC), or National Institute of Allergy and Infections Disease (NIAID)
- ❒ Local support groups
- ❒ None of the above
- ❒ Other: ___________________________

➢ ***Please continue to the next page.***

Please tell us about yourself.

1.	What is your gender?	❒ Male ❒ Female
2.	What is your race/ethnicity? Mark **all** that apply.	❒ White<br>❒ African American<br>❒ Hispanic or Latino<br>❒ Asian<br>❒ Other: ______________________
3.	What is your current marital status?	❒ Married ❒ Divorced ❒ Single
4.	What is the highest education level you have completed?	❒ Less than high school<br>❒ High school<br>❒ 2 year college<br>❒ 4 year college<br>❒ Graduate degree
5.	Which of the following categories best represents the **combined income for all family members in your household** for the past 12 months before taxes?	❒ Less than $25,000<br>❒ $25,000 - $49,999<br>❒ $50,000 - $99,999<br>❒ $100,000 - $199,999<br>❒ $200,000 - $299,999<br>❒ $300,000 or more
6.	How many children do you have under the age of 18?	❒ 1 ❒ 2 ❒ 3 ❒ 4 ❒ 5 or more
7.	Do you currently live in the same household as your food-allergic child(ren)?	❒ Yes ❒ No, (please describe how often you see your: food-allergic child(ren):_______________
8.	In the past 12 months, how many times have you spoken with a **pediatrician** during an office visit or by telephone about your child's food allergy?	❒ 0 times<br>❒ 1-2 times<br>❒ 3-5 times<br>❒ 6-9 times<br>❒ 10-12 times<br>❒ More than 12 times
9.	In the past 12 months, how many times have you spoken with an **allergist** during an office visit or by telephone about your child's food allergy?	❒ 0 times<br>❒ 1-2 times<br>❒ 3-5 times<br>❒ 6-9 times<br>❒ 10-12 times<br>❒ More than 12 times
10.	In the past 12 months, have you had to visit the emergency room (ER) for your child's food allergy?	❒ Yes, number of times: ____________________ ❒ No

➢ ***Please continue to the next page.***

Please tell us about your children under the age of 18.

	**CHILD #1**	**CHILD #2**	**CHILD #3**	**CHILD #4**
1. How old is your child?	_______ years<br>_______ months	_______ years<br>_______ months	_______ years<br>_______ months	_______ years<br>_______ months
2. What is his/her gender?	❒ Male<br>❒ Female	❒ Male<br>❒ Female	❒ Male<br>❒ Female	❒ Male<br>❒ Female
3. What is his/her race/ethnicity? Mark **all** that apply.	❒ White<br>❒ Black or African American<br>❒ Hispanic or Latino<br>❒ Asian<br>❒ Other: ________________	❒ White<br>❒ Black or African American<br>❒ Hispanic or Latino<br>❒ Asian<br>❒ Other: ________________	❒ White<br>❒ Black or African American<br>❒ Hispanic or Latino<br>❒ Asian<br>❒ Other: ________________	❒ White<br>❒ Black or African American<br>❒ Hispanic or Latino<br>❒ Asian<br>❒ Other: ________________
4. Does he/she have any of the following? Mark **all** that apply.	❒ Eczema<br>❒ Asthma<br>❒ Environmental Allergies<br>❒ None of the above	❒ Eczema<br>❒ Asthma<br>❒ Environmental Allergies<br>❒ None of the above	❒ Eczema<br>❒ Asthma<br>❒ Environmental Allergies<br>❒ None of the above	❒ Eczema<br>❒ Asthma<br>❒ Environmental Allergies<br>❒ None of the above
5. Has he/she **ever** been diagnosed with food allergy?	❒ Yes<br>❒ No**<br>****Please skip the remaining questions in the column "Child #1"**	❒ Yes<br>❒ No**<br>****Please skip the remaining questions in the column "Child #2"**	❒ Yes<br>❒ No**<br>****Please skip the remaining questions in the column "Child #3"**	❒ Yes<br>❒ No**<br>****Please skip the remaining questions in the column "Child #4"**
6. Has he/she ever experienced anaphylaxis?	❒ Yes<br>❒ No	❒ Yes<br>❒ No	❒ Yes<br>❒ No	❒ Yes<br>❒ No
7. Has he/she outgrown any food allergies?	❒ No<br>❒ Yes, please list: ________________	❒ No<br>❒ Yes, please list: ________________	❒ No<br>❒ Yes, please list: ________________	❒ No<br>❒ Yes, please list: ________________

➢ ***Please continue to the next page.***

Please tell us about your children under the age of 18 with a physician-diagnosed food allergy.

	**CHILD #1**	**CHILD #2**	**CHILD #3**	**CHILD #4**
8. Does your child have a **current** physician-diagnosed food allergy?	❒ Yes<br>❒ No**<br><br>****Please skip the remaining questions in the column "Child #1"**	❒ Yes<br>❒ No**<br><br>****Please skip the remaining questions in the column "Child #2"**	❒ Yes<br>❒ No**<br><br>****Please skip the remaining questions in the column "Child #3"**	❒ Yes<br>❒ No**<br><br>****Please skip the remaining questions in the column "Child #4"**
9. To which foods is he/she **CURRENTLY** allergic?<br><br>*Please provide the name of each food your child is allergic to and select the statements that apply to that food.*	**Food #1:** ________<br>❒ Has had a reaction to this food<br>❒ An allergy to this food has been diagnosed by a doctor<br>❒ Strictly avoiding this food	**Food #1:** ________<br>❒ Has had a reaction to this food<br>❒ An allergy to this food has been diagnosed by a doctor<br>❒ Strictly avoiding this food	**Food #1:** ________<br>❒ Has had a reaction to this food<br>❒ An allergy to this food has been diagnosed by a doctor<br>❒ Strictly avoiding this food	**Food #1:** ________<br>❒ Has had a reaction to this food<br>❒ An allergy to this food has been diagnosed by a doctor<br>❒ Strictly avoiding this food
	**Food #2:** ________<br>❒ Has had a reaction to this food<br>❒ An allergy to this food has been diagnosed by a doctor<br>❒ Strictly avoiding this food	**Food #2:** ________<br>❒ Has had a reaction to this food<br>❒ An allergy to this food has been diagnosed by a doctor<br>❒ Strictly avoiding this food	**Food #2:** ________<br>❒ Has had a reaction to this food<br>❒ An allergy to this food has been diagnosed by a doctor<br>❒ Strictly avoiding this food	**Food #2:** ________<br>❒ Has had a reaction to this food<br>❒ An allergy to this food has been diagnosed by a doctor<br>❒ Strictly avoiding this food
	**Food #3:** ________<br>❒ Has had a reaction to this food<br>❒ An allergy to this food has been diagnosed by a doctor<br>❒ Strictly avoiding this food	**Food #3:** ________<br>❒ Has had a reaction to this food<br>❒ An allergy to this food has been diagnosed by a doctor<br>❒ Strictly avoiding this food	**Food #3:** ________<br>❒ Has had a reaction to this food<br>❒ An allergy to this food has been diagnosed by a doctor<br>❒ Strictly avoiding this food	**Food #3:** ________<br>❒ Has had a reaction to this food<br>❒ An allergy to this food has been diagnosed by a doctor<br>❒ Strictly avoiding this food
	**Food #4:** ________<br>❒ Has had a reaction to this food<br>❒ An allergy to this food has been diagnosed by a doctor<br>❒ Strictly avoiding this food	**Food #4:** ________<br>❒ Has had a reaction to this food<br>❒ An allergy to this food has been diagnosed by a doctor<br>❒ Strictly avoiding this food	**Food #4:** ________<br>❒ Has had a reaction to this food<br>❒ An allergy to this food has been diagnosed by a doctor<br>❒ Strictly avoiding this food	**Food #4:** ________<br>❒ Has had a reaction to this food<br>❒ An allergy to this food has been diagnosed by a doctor<br>❒ Strictly avoiding this food

Please tell us about your children under the age of 18 with a physician diagnosed food allergy (continued).

	**CHILD #1**	**CHILD #2**	**CHILD #3**	**CHILD #4**
9. To which foods are your children **CURRENTLY** allergic? (continued)<br><br>*Please provide the name of each food your child is allergic to and select the statements that apply to that food.*	**Food #5:** ______<br>❒ Has had a reaction to this food<br>❒ An allergy to this food has been diagnosed by a doctor<br>❒ Strictly avoiding this food	**Food #5:** ______<br>❒ Has had a reaction to this food<br>❒ An allergy to this food has been diagnosed by a doctor<br>❒ Strictly avoiding this food	**Food #5:** ______<br>❒ Has had a reaction to this food<br>❒ An allergy to this food has been diagnosed by a doctor<br>❒ Strictly avoiding this food	**Food #5:** ______<br>❒ Has had a reaction to this food<br>❒ An allergy to this food has been diagnosed by a doctor<br>❒ Strictly avoiding this food
	**Food #6:** ______<br>❒ Has had a reaction to this food<br>❒ An allergy to this food has been diagnosed by a doctor<br>❒ Strictly avoiding this food	**Food #6:** ______<br>❒ Has had a reaction to this food<br>❒ An allergy to this food has been diagnosed by a doctor<br>❒ Strictly avoiding this food	**Food #6:** ______<br>❒ Has had a reaction to this food<br>❒ An allergy to this food has been diagnosed by a doctor<br>❒ Strictly avoiding this food	**Food #6:** ______<br>❒ Has had a reaction to this food<br>❒ An allergy to this food has been diagnosed by a doctor<br>❒ Strictly avoiding this food
	**Food #7:** ______<br>❒ Has had a reaction to this food<br>❒ An allergy to this food has been diagnosed by a doctor<br>❒ Strictly avoiding this food	**Food #7:** ______<br>❒ Has had a reaction to this food<br>❒ An allergy to this food has been diagnosed by a doctor<br>❒ Strictly avoiding this food	**Food #7:** ______<br>❒ Has had a reaction to this food<br>❒ An allergy to this food has been diagnosed by a doctor<br>❒ Strictly avoiding this food	**Food #7:** ______<br>❒ Has had a reaction to this food<br>❒ An allergy to this food has been diagnosed by a doctor<br>❒ Strictly avoiding this food
	**Food #8:** ______<br>❒ Has had a reaction to this food<br>❒ An allergy to this food has been diagnosed by a doctor<br>❒ Strictly avoiding this food	**Food #8:** ______<br>❒ Has had a reaction to this food<br>❒ An allergy to this food has been diagnosed by a doctor<br>❒ Strictly avoiding this food	**Food #8:** ______<br>❒ Has had a reaction to this food<br>❒ An allergy to this food has been diagnosed by a doctor<br>❒ Strictly avoiding this food	**Food #8:** ______<br>❒ Has had a reaction to this food<br>❒ An allergy to this food has been diagnosed by a doctor<br>❒ Strictly avoiding this food

~ Thank you! You have completed this survey. ~
