# Supplementary material for: Development of the Chicago Food Allergy Research Surveys: assessing knowledge, attitudes, and beliefs of parents, physicians, and the general public
Source: BMC Health Serv Res. 2009 Aug 7;9:142. doi: 10.1186/1472-6963-9-142 (PMC2736935; doi:10.1186/1472-6963-9-142)
Supplement: Additional file 2 — The Chicago Food Allergy Research Survey for Primary Care Physicians. Final validated survey instrument for pediatricians and family physicians; tool is available to all clinicians/researchers in order to (1) assess food allergy knowledge, (2) identify attitudinal barriers towards food allergy, and (3) develop targeted and effective interventions to improve the lives of food-allergic children and families. [file 1472-6963-9-142-S2.pdf]

# The Chicago Food Allergy Research Survey for the Primary Care Physicians

*The following survey is part of a study being conducted by researchers at Children's Memorial Hospital and Northwestern University Feinberg School of Medicine in Chicago, Illinois. The goal of this survey is to assess food allergy knowledge, attitudes and beliefs of the* ***pediatricians and family physicians****.*

---

**Before beginning the survey, please answer the following questions:**

1. Are you currently a practicing pediatrician or family practitioner?

   ❒ Yes

   ❒ No ⟶ We're sorry, but you are not eligible for this survey. Thank you for your interest.

2. In which state do you live?

   State: ________________________

---

**Please mark one box for each statement below.**

| | Statement | TRUE | FALSE | I DON'T KNOW |
|---|---|---|---|---|
| 1. | A positive skin prick test or RAST by itself is sufficient for the diagnosis of food allergy. | ☐ | ☐ | ☐ |
| 2. | Chronic behavioral problems may be a symptom of food allergy. | ☐ | ☐ | ☐ |
| 3. | Chronic nasal symptoms (i.e. congestion or colds that never clear up) may be a symptom of food allergy. | ☐ | ☐ | ☐ |
| 4. | Asthma is an important risk factor for severe anaphylaxis. | ☐ | ☐ | ☐ |
| 5. | Some children who have IgE-mediated milk allergies can tolerate certain yogurts and cheeses made from milk. | ☐ | ☐ | ☐ |
| 6. | A child can die from a milk allergy reaction. | ☐ | ☐ | ☐ |
| 7. | Teenagers are at higher risk for fatal food allergy compared to younger children. | ☐ | ☐ | ☐ |
| 8. | Food allergens may be passed from the maternal diet into breast milk. | ☐ | ☐ | ☐ |
| 9. | It is okay for children with egg allergies to eat egg yolks. | ☐ | ☐ | ☐ |
| 10. | Food additives (like citric acid, colorings, starch) are common food allergens. | ☐ | ☐ | ☐ |
| 11. | The number of children in the United States who have food allergies is increasing. | ☐ | ☐ | ☐ |
| 12. | Based on published research, the quality of life in children with food allergy is **better** than in children with rheumatologic disease (arthritis). | ☐ | ☐ | ☐ |
| 13. | Based on published research, the quality of life in children with food allergy is **worse** than in children with diabetes mellitus (type I). | ☐ | ☐ | ☐ |
| 14. | Taking a daily antihistamine is effective for preventing a food allergy reaction. | ☐ | ☐ | ☐ |
| 15. | There is a cure for food allergy. | ☐ | ☐ | ☐ |
| 16. | There is no absolute contraindication to prescribing self-injectable epinephrine such as EpiPen or Twinject. | ☐ | ☐ | ☐ |

➢ ***Please continue to the next page.***

17. The following are symptoms that present as ‘milk protein allergy.’ Please mark the most likely pathophysiologic mechanism that corresponds with each symptom. Choose **one** mechanism per symptom.

Blood in stool → ❒ IgE-mediated ❒ T-cell mediated ❒ Non-immune mediated ❒ I don’t know

Colic → ❒ IgE-mediated ❒ T-cell mediated ❒ Non-immune mediated ❒ I don’t know

18. A parent presents to your office with concerns that her child has milk allergy. Please mark the sign (when present independently) that is most likely to be consistent with an IgE-mediated food allergy reaction. Mark **one** answer.

❒ Flushing
❒ Hyperactivity
❒ Stomachache and bloating
❒ Headache
❒ None of the above
❒ I don’t know

19. How often do you refer your patients with food allergy to a subspecialist (allergist)?

❒ None of the time
❒ A little of the time
❒ Some of the time
❒ Most of the time
❒ All of the time

20. How often do you use a placebo demonstrator as an educational tool for the instruction of use of EpiPen or Twinject?

❒ None of the time
❒ A little of the time
❒ Some of the time
❒ Most of the time
❒ All of the time

21. Are you finding that your patients are experiencing more life-threatening reactions today as compared to five years ago?

❒ Yes
❒ No
❒ I don’t know
❒ Not applicable, I have been practicing less than 5 years

22. Are you seeing more children with food allergies today as compared to five years ago?

❒ Yes
❒ No
❒ I don’t know
❒ Not applicable, I have been practicing less than 5 years

23. Which tests would you order to diagnose food allergy? Mark **all** that apply.

❒ Food specific IgG levels
❒ Food specific IgE levels
❒ Oral food challenges
❒ Desensitization
❒ Skin-prick test
❒ None of the above
❒ I don’t know

24. Which of the following in isolation may be a symptom of food allergy in an infant? Mark **all** that apply.

❒ Colic
❒ Fussiness
❒ Hives
❒ Spitting up
❒ Chronic diarrhea
❒ Eczema
❒ None of the above
❒ I don’t know

25. Which of the following are medical indication(s) to change an infant’s formula? Mark **all** that apply.

❒ Colic
❒ Fussiness
❒ Hives
❒ Spitting up
❒ Chronic diarrhea
❒ Eczema
❒ None of the above
❒ I don’t know

26. Which of the following are the **three** most common allergies in children? Mark three answers.

❒ Egg
❒ Milk
❒ Soy
❒ Tomato
❒ Wheat
❒ Citrus *(orange, lemon, grapefruit, lime)*
❒ Peanut
❒ Shellfish *(shrimp, lobster, crab)*
❒ Strawberry
❒ Tree nuts *(almonds, walnuts, pecans, cashews)*

27. Which of the following contain food ingredients that could cause an allergic reaction? Mark all that apply.

❒ Play-doh
❒ Medicines
❒ Toothpaste
❒ Lotions & creams

28. Which food component causes allergic reactions? Mark the best answer.

- ❒ Protein
- ❒ Fat
- ❒ Carbohydrate
- ❒ None of the above
- ❒ I don't know

29. Which age group is most likely to have food allergies?

- ❒ 0-5 years
- ❒ 6-10 years
- ❒ 11-15 years
- ❒ Greater than 15 years
- ❒ I don't know

30. What percent of children with **milk** allergy develop tolerance (or outgrow the allergy)?

- ❒ Nearly 0%
- ❒ Less than 25%
- ❒ Between 25% - 50%
- ❒ Between 51% - 75%
- ❒ Greater than 75%
- ❒ I don't know

31. What percent of children with **peanut** allergy develop tolerance (or outgrow the allergy)?

- ❒ Nearly 0%
- ❒ Less than 25%
- ❒ Between 25% - 50%
- ❒ Between 51% - 75%
- ❒ Greater than 75%
- ❒ I don't know

32. What percent of moderate to severe **atopic dermatitis** is associated with food allergy?

- ❒ Nearly 0%
- ❒ Less than 25%
- ❒ Between 25% - 50%
- ❒ Between 51% - 75%
- ❒ Greater than 75%
- ❒ I don't know

33. How often does timely administration of epinephrine prevent fatal anaphylaxis?

- ❒ None of the time
- ❒ A little of the time
- ❒ Some of the time
- ❒ Most of the time
- ❒ All of the time
- ❒ I don't know

34. Which of the following vaccines are not safe for children with egg allergy? Mark **all** that apply.

- ❒ Influenza
- ❒ Varicella (Chickenpox)
- ❒ Measles Mumps Rubella (MMR)
- ❒ I don't know

35. Which of the following doses of epinephrine would you prescribe to a child who weighs 60 lbs (27 kg)?

- ❒ EpiPen Jr. or Twinject Jr. (0.15 mg of epinephrine)
- ❒ EpiPen or Twinject (0.3 mg of epinephrine)
- ❒ Neither of the above
- ❒ I don't know

36. Which location do you instruct your patients to inject epinephrine autoinjectors (EpiPen or Twinject)?

- ❒ Upper arm
- ❒ Lateral thigh
- ❒ Buttock
- ❒ None of the above
- ❒ I don't know

37. Which of the following are evidence-based recommendations as preventative measures for children at high-risk of developing food allergy? Mark **all** that apply.

- ❒ Delaying introduction of specific foods
- ❒ Withholding solids until 4 months of age
- ❒ Switching from a cow's milk to soy based formula
- ❒ I don't know

38. A peanut-allergic child presents to your office after ingestion of peanuts 30 minutes ago. He has diffuse urticarial lesions on his trunk and face and periorbital swelling. Shortly after arrival to your office, he develops repetitive emesis followed by a persistent cough. His vital signs are stable and his exam reveals clear breath sounds but decreased air movement. He is 25 kg. What is the first treatment that you would give to this patient? Mark the best answer.

- ❒ Albuterol nebulized treatment 2.5 mg
- ❒ Diphenhydramine 25 mg orally
- ❒ Epinephrine 1:1,000 intramuscular
- ❒ Epinephrine 1:1,000 subcutaneus
- ❒ Prednisone 25 mg orally
- ❒ I don't know

39. The Food Allergen Labeling and Consumer Protection Act (FALCPA), which took effect January 1, 2006, mandates that:

- ❒ Foods containing milk, eggs, fish, crustacean shellfish, peanuts, tree nuts, wheat, and soy must declare the food in plain language on the ingredient list
- ❒ The label must list the FDA-certified color additives by name
- ❒ Ingredients must be listed in descending order by weight
- ❒ None of the above
- ❒ I don't know

40. Which of the following do you think is the most important to help people with food allergies?

❒ Develop a cure for food allergy
❒ Improve the treatments of food allergy
❒ Identify the causes of food allergy
❒ Promote school education programs about food allergy
❒ Promote public awareness campaigns about food allergy

**Please mark one box for each statement below.**

| | **Strongly Disagree** | **Disagree** | **Neither Agree nor Disagree** | **Agree** | **Strongly Agree** |
|---|---|---|---|---|---|
| 41. Most people take food allergy seriously. | ☐ | ☐ | ☐ | ☐ | ☐ |
| 42. Parents of children with food allergy are hypervigilant. | ☐ | ☐ | ☐ | ☐ | ☐ |
| 43. I feel comfortable interpreting laboratory tests to establish a diagnosis of food allergy. | ☐ | ☐ | ☐ | ☐ | ☐ |
| 44. My medical training adequately prepared me to care for food allergy patients. | ☐ | ☐ | ☐ | ☐ | ☐ |
| 45. I am confident in my ability to care for patients with food allergy. | ☐ | ☐ | ☐ | ☐ | ☐ |
| 46. I feel confident that the staff in the **local emergency department** will discharge patients who've had food allergy reactions with an epinephrine autoinjector (EpiPen or Twinject). | ☐ | ☐ | ☐ | ☐ | ☐ |
| 47. All classrooms should be nut-free (including peanuts and tree nuts). | ☐ | ☐ | ☐ | ☐ | ☐ |
| 48. In schools, EpiPen/Twinject should be kept only in nurse/principal/counselor's offices. | ☐ | ☐ | ☐ | ☐ | ☐ |
| 49. In comparison to federal funding levels of $400 million per year for asthma research and $1.2 billion per year for diabetes research, **$10 million per year for food allergy research** is sufficient. | ☐ | ☐ | ☐ | ☐ | ☐ |

| | **Strongly Disagree** | **Disagree** | **Neither Agree nor Disagree** | **Agree** | **Strongly Agree** | **Does Not Apply** |
|---|---|---|---|---|---|---|
| 50. Physicians in a group practice: My partners are knowledgeable about food allergy. | ☐ | ☐ | ☐ | ☐ | ☐ | ☐ |

➢ ***Please continue to the next page.***

**Please tell us about yourself.**

| | | |
|---|---|---|
| 1. | What is your medical specialty? | ❒ Pediatrics ❒ Family Practice ❒ Other: _____________ |
| 2. | In what year did you complete your residency? | __ __ __ __ |
| 3. | How would you describe your practice? Choose **one** answer that best fits. | ❒ Practicing clinician affiliated with an academic center<br>❒ Private practice clinician in the community<br>❒ Hospital-based clinician<br>❒ Other: ____________________ |
| 4. | In which zip code is your practice located? | __ __ __ __ __ |
| 5. | If you see children in an outpatient setting, on average about how many children (0-18 years) do you see per week? | ________ children |
| 6. | Please estimate the percentage of your patient population that fall into each **insurance** category. The total must equal 100%. | ____% Employer-based/Private insurance<br>____% Medicaid or other state-funded programs<br>____% Uninsured<br>____% Unknown |
| 7. | Please estimate the percentage of your patient population that fall into each **racial/ethnic** category. The total must equal 100%. | ____% White<br>____% Black/African American<br>____% Hispanic/Latino<br>____% Asian<br>____% Other: __________________<br>____% Unknown |
| 8. | What percentage of your patients have food allergy? | ❒ None ❒ 6-10%<br>❒ Less than 5% ❒ More than 10% |
| 9. | Please estimate the percentage of your **food allergy** patient population that fall into each racial/ethnic category. This total must equal 100%. | ____% White<br>____% Black/African American<br>____% Hispanic/Latino<br>____% Asian<br>____% Other: __________________<br>____% Unknown |
| 10. | Do any of the following people have a food allergy? | ❒ Me<br>❒ Spouse/partner or child<br>❒ Relative or close friend<br>❒ None of the above |

***~ Thank you! You have completed this survey. ~***
