# Supplementary material for: Development of the Chicago Food Allergy Research Surveys: assessing knowledge, attitudes, and beliefs of parents, physicians, and the general public
Source: BMC Health Serv Res. 2009 Aug 7;9:142. doi: 10.1186/1472-6963-9-142 (PMC2736935; doi:10.1186/1472-6963-9-142)
Supplement: Additional file 3 — The Chicago Food Allergy Research Survey for the General Public. Final validated survey instrument for the general public; upon publication, tool is available to all clinicians/researchers in order to (1) assess food allergy knowledge, (2) identify attitudinal barriers towards food allergy, and (3) develop targeted and effective interventions to improve the lives of food-allergic children and families. [file 1472-6963-9-142-S3.pdf]

The Chicago Food Allergy Research Survey for the General Public

*The following survey is part of a study being conducted by researchers at Children's Memorial Hospital and Northwestern University Feinberg School of Medicine in Chicago, Illinois. The goal of this survey is to assess food allergy knowledge, attitudes and beliefs of the **general public**.*

Before beginning the survey, please answer the following questions:

1. Do you know anyone with a food allergy?

   ❒ No

   ❒ Yes (Mark *all* that apply):
   - ❒ Me
   - ❒ Child ages 0-18 → 1a. Does your child have a current food allergy that has been diagnosed by a doctor?
     - ❒ No
     - ❒ Yes → We're sorry, but you are not eligible for this survey. Thank you for your interest.
   - ❒ Spouse/partner
   - ❒ Friend or relative
   - ❒ Child's classmate or friend

2. Are you a pediatrician or a family practitioner?

   ❒ Yes

   ❒ No → We're sorry, but you are not eligible for this survey. Thank you for your interest.

3. In which state do you live?

   State: ________________________

Please mark one box for each statement below.

	TRUE	FALSE	I DON'T KNOW
1. An allergic reaction can happen when the body considers a food to be harmful.	☐	☐	☐
2. Lactose intolerance (trouble digesting dairy products) is the same as having a milk allergy.	☐	☐	☐
3. A person can die from having a food allergy reaction.	☐	☐	☐
4. Hives (red bumps or blotches on the skin that can be itchy) are a <u>common</u> symptom of a food allergy reaction.	☐	☐	☐
5. People with food allergies can have an allergic reaction after touching a food.	☐	☐	☐
6. A person with a milk allergy can still drink low-fat milk without having an allergic reaction.	☐	☐	☐
7. Foods eaten by a mother can be passed to her child through her breast milk.	☐	☐	☐
8. Acidic foods (like lemons, oranges, and tomatoes) <u>commonly</u> cause food allergy.	☐	☐	☐
9. Allergic diseases run in families.	☐	☐	☐
10. Food allergies can go away as a person gets older.	☐	☐	☐
11. Food allergy is more common in children than adults.	☐	☐	☐
12. The number of children in the United States who have a food allergy has been increasing over the past ten years.	☐	☐	☐
13. There is a cure for food allergy.	☐	☐	☐
14. The <u>only</u> way to prevent an allergic reaction is to stay away from the food that causes the allergy.	☐	☐	☐
15. A person can take a medicine everyday to prevent having food allergy reactions.	☐	☐	☐
16. There is a law in the United States that requires all foods to be labeled with allergy information.	☐	☐	☐

17. Which of the following are the <u>**three**</u> most common food allergies in <u>**children**</u>? Mark <u>*three*</u> answers.

❐ Egg
❐ Wheat
❐ Milk
❐ Peanut
❐ Tree nuts *(almonds, walnuts, pecans, cashews)*
❐ Shellfish *(shrimp, lobster, crab)*

18. Which of the following is the most common food allergy in <u>**adults**</u>? Mark <u>*one*</u> answer.

❐ Milk
❐ Peanut
❐ Shellfish *(shrimp, lobster, crab)*
❐ I don't know

19. A boy with a milk allergy accidentally drank some milk. Please mark which of the following could be a sign of food allergy reaction. Mark *all* that apply.

- ❐ After 2 days he gets hyperactive and cranky and has headaches
- ❐ After 15 minutes he gets hives on his face and chest
- ❐ Immediately his tongue swells and he has trouble breathing
- ❐ He has a stuffy nose that won't go away for weeks

Please mark one box for each statement below.

	Strongly Disagree	Disagree	Neither Agree nor Disagree	Agree	Strongly Agree
20. Food allergy is a serious health problem in the United States.	☐	☐	☐	☐	☐
21. People with food allergies are treated differently because of their food allergy.	☐	☐	☐	☐	☐
22. Children with food allergies have overprotective parents.	☐	☐	☐	☐	☐
23. Children with food allergies are teased at school.	☐	☐	☐	☐	☐
24. For someone who has a food allergy, staying away from the food that he or she is allergic to is difficult.	☐	☐	☐	☐	☐
25. People with food allergies worry a lot about their allergy.	☐	☐	☐	☐	☐
26. It is difficult for people with food allergies to safely eat at restaurants.	☐	☐	☐	☐	☐
27. Having an EpiPen or Twinject (injectable epinephrine) is important for most children with severe food allergies.	☐	☐	☐	☐	☐
28. Schools should have plans for keeping children with food allergies safe at school.	☐	☐	☐	☐	☐

29. Which of the following do you think is the most important to help people with food allergies? Mark *one* answer.

- ❐ Develop a cure for food allergy
- ❐ Improve the treatments of food allergy
- ❐ Find the causes of food allergy
- ❐ Promote school education programs for food allergy
- ❐ Promote public awareness campaigns for food allergy

30. Which of the following would be the best way to learn about food allergy? Mark *one* answer.

- ❐ Radio
- ❐ Television (TV)
- ❐ Handout/Brochure
- ❐ Internet/Email
- ❐ Newspapers/Magazines
- ❐ Other: ____________________

Before continuing, please answer the following questions:

1. Do you have children under the age of 18?

❒ No → Please skip to the next page.

❒ Yes → Please continue to the next question.

2. Do your children attend any of the following? Mark *all* that apply.

❒ No children
❒ No children in school
→ Please skip to the next page.

❒ Preschool
❒ Elementary School
❒ Middle School
❒ High School
→ Please continue to the next question.

Please mark one box for each statement below.

	**Strongly Disagree**	**Disagree**	**Neither Agree nor Disagree**	**Agree**	**Strongly Agree**
31. Schools should ban all products with nuts.	☐	☐	☐	☐	☐
32. Schools should have special tables where children with food allergies can safely eat lunch.	☐	☐	☐	☐	☐
33. It would be unfair if my child could not have a peanut butter sandwich because of another student's peanut allergy.	☐	☐	☐	☐	☐
34. I would worry about having a child with food allergy play at my house.	☐	☐	☐	☐	☐

35. What would be the best way for schools to teach parents about how to protect children with food allergies? Mark *one* answer.

❒ Handouts/brochures in the mail
❒ Presentation at parent-teacher meetings
❒ Parents of food-allergic children talking to other parents
❒ Doctor or nurse talking about food allergies
❒ Other: ________________________

➢ ***Please continue to the next page.***

Please tell us about yourself.

1.	How old are you?	❒ 18 - 24 ❒ 25 – 44<br>❒ 45 - 65 ❒ Over 65
2.	What is your gender?	❒ Male ❒ Female
3.	What is your race/ethnicity? Mark ***all*** that apply.	❒ White<br>❒ African American<br>❒ Hispanic or Latino<br>❒ Asian<br>❒ Other: __________________
4.	What is the highest education level you have completed?	❒ Less than high school ❒ 4 year college<br>❒ High school ❒ Graduate degree<br>❒ 2 year college
5.	Which of the following categories best represents the **combined income for all family members in your household** for the past 12 months before taxes?	❒ Less than $25,000 ❒ $75,000 - $99,999<br>❒ $25,000 - $49,999 ❒ $100,000 - $149,999<br>❒ $50,000 - $74,999 ❒ $150,000 or more
6.	Have you had any experience or training with with food allergy through your job or work?	❒ Yes ❒ No

~ Thank you! You have completed this survey. ~
